# Anti-Cancer Effect of Lambertianic Acid by Inhibiting the AR in LNCaP Cells

**DOI:** 10.3390/ijms17071066

**Published:** 2016-07-07

**Authors:** Myoung-Sun Lee, Seon-Ok Lee, Sung-Hoon Kim, Eun-Ok Lee, Hyo-Jeong Lee

**Affiliations:** Department of Cancer Preventive Material Development, Graduate School, Kyung Hee University, 1 Hoegi-dong, Dongdaemun-gu, Seoul 130-701, Korea; lmsms14@naver.com (M.-S.L.); lso4595@naver.com (S.-O.L.); sungkim7@khu.ac.kr (S.-H.K.); leook@khu.ac.kr (E.-O.L.)

**Keywords:** LNCaP, lambertianic acid, androgen receptor, anticancer

## Abstract

Lambertianic acid (LA) is known to have anti-allergic and antibacterial effects. However, the anticancer activities and mechanism of action of LA have not been investigated. Therefore, the anticancer effects and mechanism of LA are investigated in this study. LA decreased not only AR protein levels, but also cellular and secretory levels of PSA. Furthermore, LA inhibited nuclear translocation of the AR induced by mibolerone. LA suppressed cell proliferation by inducing G_1_ arrest, downregulating CDK4/6 and cyclin D1 and activating p53 and its downstream molecules, p21 and p27. LA induced apoptosis and the expression of related proteins, including cleaved caspase-9 and -3, c-PARP and BAX, and inhibited BCl-2. The role of AR in LA-induced apoptosis was assessed by using siRNA. Collectively, these findings suggest that LA exerts the anticancer effect by inhibiting AR and is a valuable therapeutic agent in prostate cancer treatment.

## 1. Introduction

Prostate cancer is the second most common form of cancer in males and a direct cause of death in men worldwide [1,2]. The androgen receptor (AR) function is important in sexual and physiological development [3]. In particular, androgen and the AR are important for prostate development [4]. The AR, the role of which was demonstrated in the late 1960s, conducts an important role in the growth and proliferation of androgen-dependent prostate cancer and cells [5,6,7]. Testosterone and dihydrotestosterone exert their biological effects by binding to the AR and inducing transcriptional activity [6]. AR regulates the prostate-specific antigen (PSA) expression in prostate cancer cells [8,9]. PSA is considered the most sensitive biomarker available for confirming the existence of prostatic disease, prostate cancer [10]. Previous studies show that anti-spermatogenesis, anticancer activity and apoptosis in prostate cancer cells were induced by the suppression of AR signaling [11,12,13]. Furthermore, several studies have reported that p53 regulates the AR, transcription of PSA [14] and induces AR downregulation in prostate cancer [15]. The suppression of anti-apoptosis protein expression by AR-mediated androgen action has been demonstrated as a mechanism for stimulating anti-apoptosis protein expression during androgen deprivation [16]. Lambertianic acid was originally isolated and identified from *Pinus* *koraiensis* *Siebold* and *Zucc* (Pinaceae) [17]; our previous studies showed that it exerts anti-obesity effects [18]. LA is known to exert hepatoprotective, hemopoiesis-stimulatory and neurotropic activities [19]. However, its anticancer activity has not been investigated. Therefore, the purpose of the present study was to investigate the anticancer activity of LA likely mediated via the AR pathway in LNCaP cells.

## 2. Results

### 2.1. Lambertianic Acid Inhibits Cell Growt

**Figure 1:**
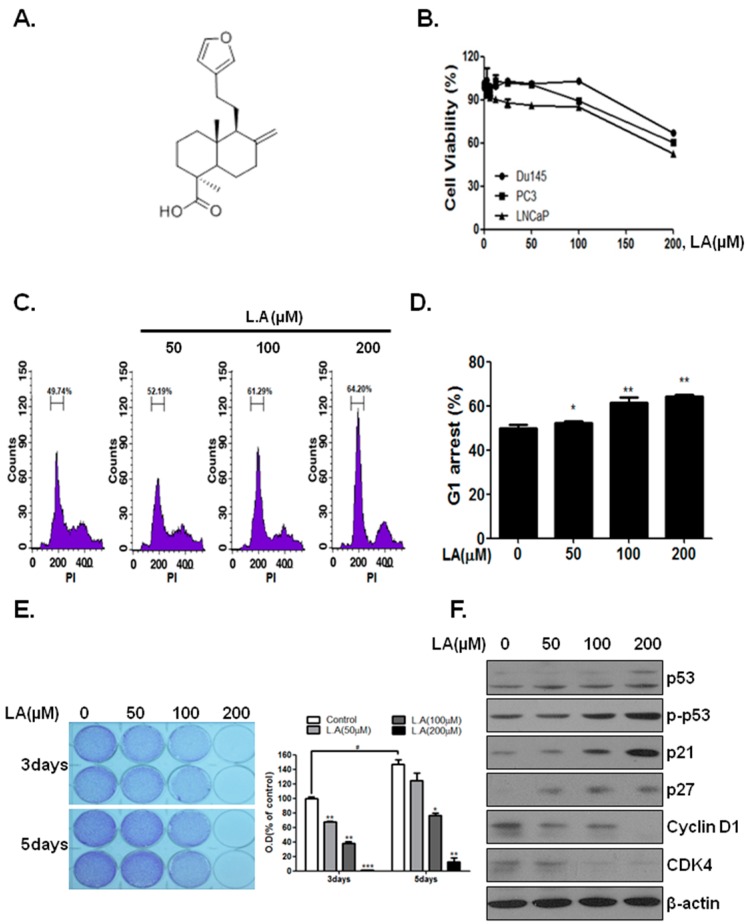
Effect of LA on induced G_1_ arrest and proliferation after 24 h of incubation with LNCaP cells. (**A**) Chemical structure of LA; (**B**) Cytotoxicity of LA against prostate cancer cells was determined by the MTT assay. Cells were treated with various concentrations of LA (0, 3.125, 6.25, 12.5, 25, 50, 100 and 200 μM) for 24 h; (**C**) LNCaP cells were treated with LA (0, 50, 100 and 200 µM) for 24 h and stained with propidium iodide (PI) after fixation. Stained cells were analyzed using a FACS Vantage flow cytometry system; (**D**) G_1_ arrest (%) in LA-treated cell after 24 h. * *p* < 0.05 and ** *p* < 0.01 (in comparison to the control); (**E**) Anti-proliferative activity of LA was evaluated using the cell growth assay. LNCaP cells were treated with LA (0, 50, 100, 200 μM) and incubated for three and five days. Cells were stained; then, randomly chosen fields were photographed and resolved in 70% ethanol after washing with distilled water, and absorbance was read using a microplate reader. # *p* < 0.05, * *p* < 0.05, ** *p* < 0.01 and *** *p* < 0.001 (in comparison to the control); (**F**) LNCaP cells were treated with LA (0, 50, 100 and 200 µM) for 24 h and subjected to Western blot analysis of protein levels (p-P53, P53, P21, P27, cyclin D1 and CDK4).

LNCaP cells were affected more than castration-resistant cells (PC-3 and DU145) by LA (Figure 1B). Incubation with 200 μM and 400 μM (data not shown) LA for 24 h reduced LNCaP cell viability by 35% and 92.2% (data not shown), respectively, as compared to the control. The growth inhibition was accompanied by G_1_ phase arrest (Figure 1C,D). To determine whether LA inhibits cancer cell proliferation following a longer exposure, LNCaP cells were treated with LA (0, 50, 100 and 200 µM) for three and five days, and then, cell proliferation was examined using crystal violet staining. As shown in Figure 1E, LA decreased the number of LNCaP cells concentration and time dependently (IC_50_ 109 μM). To determine whether LA affects the expression level of cell proliferation-related proteins, proteins were analyzed using Western blotting. LA treatment for 24 h decreased the G_1_ regulat,dicate that the suppression of cell proliferation by LA was mediated by changes in related protein levels.

### 2.2. Lambertianic Acid Induces the Apoptosis of LNCaP Cells

**Figure 2:**
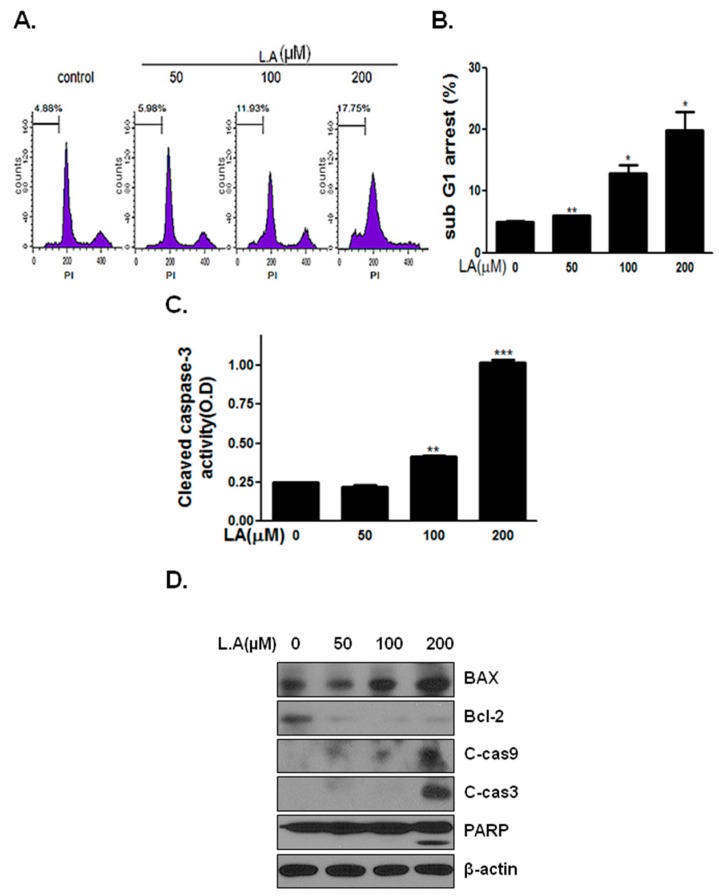
Effect of LA on induced apoptosis after 48 h of incubation with LNCaP cells. (**A**) LNCaP cells were treated with LA (0, 50, 100 and 200 µM) for 48 h and then stained with propidium iodide (PI) after fixation. Stained cells were analyzed using a FACS Vantage flow cytometry system. Analysis of the cell cycle showed increased sub-G_1_; (**B**) Sub-G_1_ arrest (%) in LA-treated cells after 48 h. * *p* < 0.05 and ** *p* < 0.01 (in comparison to the control); (**C**) Cleaved caspase-3 activity was measured following the treatment of LNCaP cells with LA (0, 50, 100 and 200 µM) for 48 h. ** *p* < 0.01 and *** *p* < 0.001 (in comparison to the control); (**D**) LNCaP cells were treated with LA (0, 50,100 and 200 µM) for 48 h and subjected to Western blot analysis of apoptosis marker protein levels (Bcl-2, BAX, cleaved caspase-9, cleaved caspase-3, PARP).

As shown in Figure 2A,B, LA treatment for 48 h induced the sub-G_1_ phase for the concentrations of LNCaP cells. To determine the potential molecular mediators of the apoptotic effects, the caspase cleavage patterns, PARP cleavage, Bcl-2 and BAX protein levels were analyzed. LA enhanced cleaved caspase-3 activity (Figure 2C). LA increased cleaved caspase-3 and caspase-9 levels at a 200-µM concentration, which corresponded to the increase in PARP cleavage (Figure 2D). Furthermore, LA induced the mitochondrial death mediator protein, BAX, and inhibited Bcl-2.

### 2.3. Lambertianic Acid Attenuates AR and PSA Expression in LNCaP Cells

**Figure 3:**
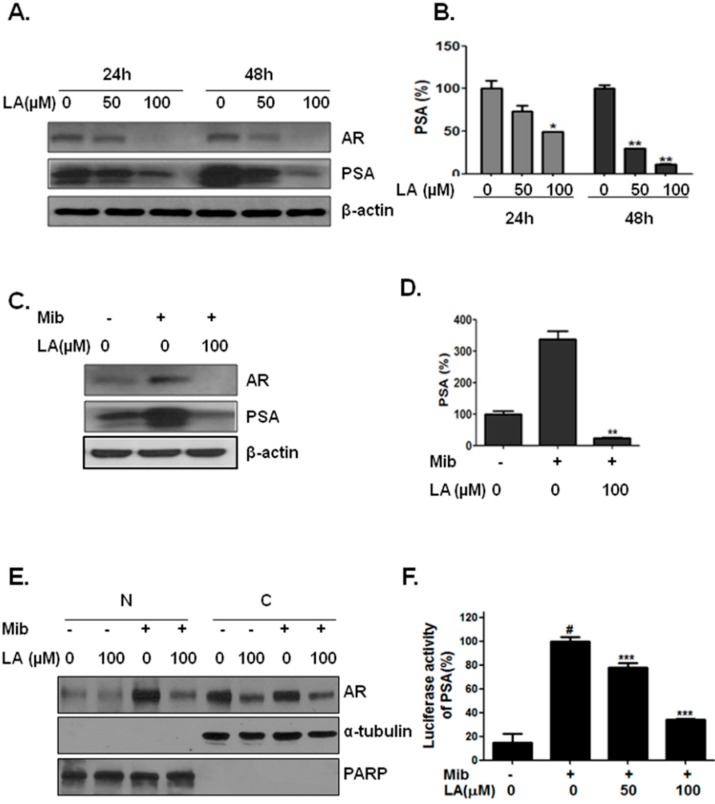
Concentration-dependent inhibition of PSA and AR and the time course of inhibition of PSA and AR by LA. (**A**) Western blot analysis of cellular prostate-specific antigen (PSA) and androgen receptor (AR) following the treatment of LNCaP cells with LA for 24 and 48 h; (**B**) ELISA measurement of secreted PSA after 24 h. * *p* < 0.05 and ** *p* < 0.01 (in comparison to the control); (**C**) LNCaP cells were seeded in phenol red-free medium supplemented with 5% charcoal-stripped serum. Western blot analyses of cellular PSA and AR following treatment with LA, with or without mibolerone (Mib); (**D**) LNCaP cells were seeded in phenol red-free medium supplemented with 5% charcoal-stripped serum. ELISA of secreted PSA measured following treatment with LA, with or without Mib. ** *p* < 0.01 (in comparison to the control); (**E**) LNCaP cells were seeded in phenol red-free medium supplemented with 5% charcoal-stripped serum in a T25 plate. Cells were treated with LA (0 and 100 µM) and Mib (1 nM). LNCaP cells were harvested and then separated into nuclear and cytosolic fractions using the NE-PER Nuclear and Cytoplasmic Extraction Reagent kit (Thermo Scientific, Waltham, MA, USA), and then, the specificity of separation was confirmed using Western blotting for total poly-ADP-ribose polymerase (PARP) and α-tubulin as respective nuclear and cytosolic markers; (**F**) LNCaP cells were seeded in phenol red-free medium supplemented with 5% charcoal-stripped serum in culture plates and then transfected with luciferase reporter plasmids combined with the PSA-Luc reporter for 24 h. Cells were treated with LA (0, 50 nd 100 µM) or mibolerone (Mib, 1 nM) and lysed, and then reporter activity was analyzed using the Luciferase Reporter Assay system (Promega, Madison, AL, USA). # *p* < 0.05 (in comparison to the untreated control) and *** *p* < 0.001 (in comparison to the Mib-treated control).

The effect of a non-apoptotic concentration of LA was tested on PSA and AR expression after treatment for 24 and 48 h. As shown in Figure 3A, LA decreased the PSA and AR protein level following 24 and 48 h of exposure. Furthermore, LA decreased the secretion of PSA into the conditioned medium concentration and time dependently (Figure 3B). Incubation with 100 μM LA for 24 h and 48 h led to a 51% and a 90% reduction, respectively.

### 2.4. Lambertianic Acid Inhibits Androgen-Stimulated AR Nuclear Translocation

To determine whether LA affects the AR and PSA level of androgen-stimulated LNCaP cells, they were pretreated with LA (0 and 100 µM) for 1 h and then further stimulated with mibolerone (Mib, 1 nM) for 23 h in the presence of LA. As shown in Figure 3C, LA decreased the AR protein level, while Figure 3C,D shows that in the presence of Mib, LA effectively blocked the androgen-stimulated cellular and secreted PSA.

To determine whether LA affects AR nuclear translocation, LNCaP cells were pretreated with LA (0 and 100 µM) for 1 h and then further stimulated with Mib (1 nM) for 23 h in the presence of LA. As shown in Figure 3E, treatment with LA blocked the androgen-stimulated translocation of AR to the nucleus and decreased its protein expression. These results suggest that LA prevented AR from activating PSA mRNA transcription and protein expression. To verify the transcriptional inhibition induced by LA, the PSA promoter luciferase activity was evaluated by a transient transfection assay. LA treatment for 24 h inhibited the androgen-stimulated PSA promoter transcription concentration dependently (Figure 3F).

### 2.5. AR Signaling Mediates Lambertianic Acid-Induced Cell Proliferation Suppression and Apoptosis

**Figure 4:**
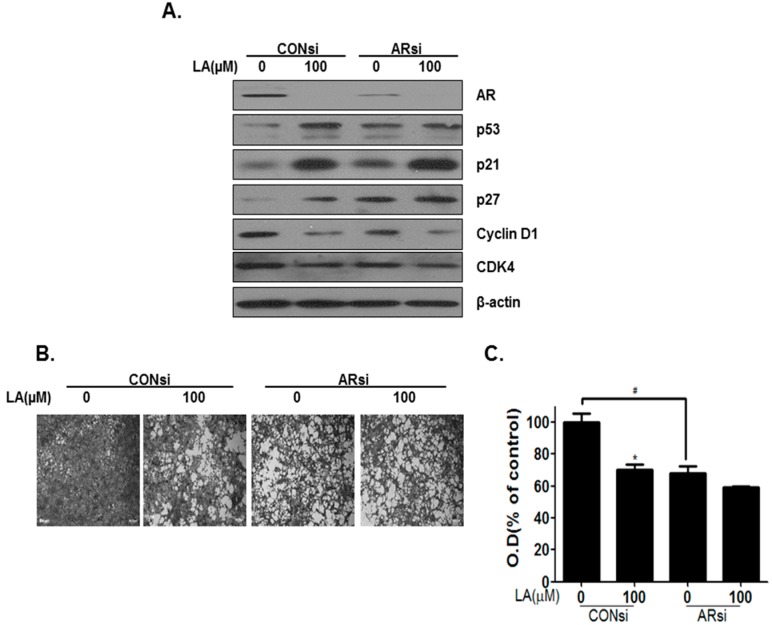
Effect of silencing androgen receptor (AR)-induced proliferation in LNCaP cells. (**A**) LNCaP cells were transfected with AR-siRNA for 24 h, treated with LA (0 and 100 µM) for 24 h and then analyzed using Western blot; (**B**) Anti-proliferative activity following silencing of AR was evaluated using the cell growth assay. LNCaP cells were transfected with AR siRNA for 24 h, treated with LA (0 and 100 µM), and incubated for three days; (**C**) Cells were stained; randomly chosen fields were photographed and resolved in 70% ethanol after washing with distilled water, and absorbance was read using a microplate reader (590 nm). # *p* < 0.05 and * *p* < 0.05 (in comparison to the control).

**Figure 5:**
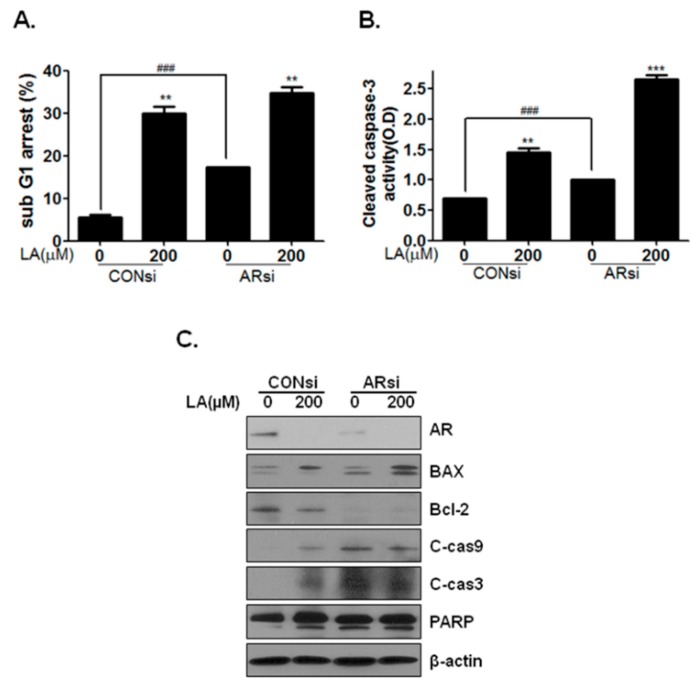
Effect of silencing androgen receptor (AR)-induced apoptosis of LNCaP cells. (**A**) Cell cycle analysis was measured following treatment with LA (0 and 200 µM) for 48 h after transfection with AR-siRNA for 24 h in LNCaP cells. ### *p* < 0.001 and ** *p* < 0.01 (in comparison to the control); (**B**) Cleaved caspase-3 activity also was measured with treated LA (0 and 200 µM) for 48 h after transfection with AR-siRNA for 24 h in LNCaP cells; (**C**) LNCaP cells were transfected with AR-siRNA for 24 h, treated with LA (0 and 200 µM) for 48 h and then subjected to Western blot analysis of apoptosis-related protein expression. ### *p* < 0.001, ** *p* < 0.01 and *** *p* < 0.001 (in comparison to the control).

This study showed that AR is a pivotal factor in prostate cancer development. The inhibitory effects of LA on AR were confirmed by transfecting AR siRNA into LNCaP cells. Silencing AR reduced its level, as well as that of PSA in the presence or absence of LA. Furthermore, AR knockdown inhibited G_1_ regulatory protein levels (cyclin D1 and CDK4) and increased the tumor suppressor genes (p53, p21 and p27) (Figure 4A). In addition, knockdown of the AR in LNCaP cells suppressed their proliferation by 30.1% compared to cells transfected with the CON siRNA (Figure 4B,C). Accordingly, these results demonstrated that inhibition of AR expression by AR siRNA suppressed the proliferation of LNCaP cells. To confirm apoptosis by silencing AR, cell cycle and cleaved caspase-3 activity analyses were performed. AR knockdown increased the sub-G_1_ phase and caspase-3 activity (Figure 5A,B). In addition, AR silencing increased apoptosis-related protein expression (BAX, cleaved caspase-9, cleaved caspase-3 and cleaved-PARP) and inhibited Bcl-2 (Figure 5B). These results suggest that AR plays an important role in LA-induced cancer-cell growth inhibition in AR-sensitive LNCaP cells.

## 3. Discussion

Here, we demonstrated that LA blocked AR signaling (anti-androgenic) by impairing the nuclear translocation of the stimulated AR and decreasing its protein expression. LA is a bioactive diterpene, which exerts anti-allergic and antibacterial effects [17,18]. LA is found to naturally occur in Pinaceae or Cupressaceae family species [20,21]. We recently reported that LA, a bioactive constituent of *P. koraiensis*, has anti-obesity effects [22]. Nevertheless, the specific effect of LA on tumor proliferation and survival has not been examined previously. Our findings indicate that LA has anticancer effects in AR-dependent prostate cancer cells, as revealed by the determination of AR regulation. In this study, LA suppressed AR expression, as well as cellular and secretory levels of PSA in AR-sensitive cells. Androgen and the AR are crucial for prostate function and are involved in various stages of diseases [4].

PSA is a gene that is regulated by androgen in the normal prostate and prostate cancer cells [23]. PSA is a biomarker routinely used for the early detection of prostate cancer and in monitoring response to treatments [24]. The AR plays a pivotal role in prostate cancer development [6,7] and is related to cell cycle progression in the G_1_/S phase [25], as well as inhibition of apoptosis. Several studies have reported that natural compounds or herbal extracts inhibit cancer cell growth and induce apoptosis by downregulating AR signaling [26,27].

Inhibition of the AR suppresses cell cycle progression by inhibiting the function and activity of cyclins and CDKs in androgen-dependent prostate cancer cells [28]. Cyclin/CDK complexes are important regulators of the cell cycle and are involved in abnormal cancer cell growth [29]. Treatment of LNCaP cells with LA for 24 h increased the G_1_ phase by downregulating CDK and cyclin D1 (Figure 1F), while treatment for 48 h induced apoptosis (Figure 2D).

To verify the role of AR signaling in the anticancer effects of LA, its role in the anti-proliferation and apoptosis induced by LA was examined using AR siRNA transfection and Mib. Mib is a potent anabolic steroid and has a high affinity for the androgen receptor. Overexpression of AR with Mib induced cell proliferation-related proteins cyclinD1 and PCNA and inhibited apoptosis by inducing BCl-2 and cleaved PARP, while LA inhibited Mib-induced cell proliferation and induced apoptosis in LNCaP cells with Mib (Supplementary Figure S1). The deficiency of AR increased the G_1_ phase in the prostate cancer cells [25]. Liao et al. [30] reported that siRNA-induced AR silencing leads to apoptotic cell death in prostate cancer. Consistently, our data showed that AR siRNA transfection noticeably induced apoptosis by upregulating BAX, cleaved caspase-9, -3 and cleaved PARP and downregulating Bcl-2. Furthermore, our results showed that AR siRNA transfection decreased cell proliferation by downregulating CDK and cyclin D1, as well as upregulating p21 and p27. Knockdown of AR with siRNA enhanced the inhibitory effect of LA on LNCaP cell proliferation by inhibiting cyclin D1 and CDK4 and inducing p53, p21 and p27 (Figure 4). Further, AR knockdown enhanced LA-mediated apoptosis by inducing BAX and cleaved caspase-9, -3 and PARP and inhibiting BCl-2, as well as inducing subG1 arrest and cleaved caspase-3 activity. Combined, these data suggest that LA suppresses cell proliferation and induces apoptosis via the inhibition of AR signaling in androgen-responsive prostate cancer cells.

LA affects more LNCaP cells than PC-3 and DU145 cells in Figure 1B. However, 200 μM LA has a cytotoxic effect on even castration-resistant cells, both PC-3 and DU145 cells. Furthermore, knockdown of AR using AR siRNA enhances the LA-induced apoptosis (Figure 5). These data suggest that LA has the potential to inhibit upstream proteins of AR, such as NF-kappaB [31], MAPKs (AKT/PKB, PKA, PKC) and STAT-3 [32] or other androgen receptor coregulators. 

In summary, LA suppressed protein expression and nuclear translocation of AR in androgen-stimulated LNCaP cells. In addition, LA significantly reduced the secretory and cellular levels of PSA in a concentration-dependent manner. LA suppressed cell proliferation by inhibiting G_1_ arrest and induced apoptosis by inhibiting AR signaling. Moreover, silencing AR siRNA blocked the stimulation of cell proliferation and enhanced apoptosis. This study elucidated the anticancer mechanism of LA by demonstrating that this effect was mediated via inhibition of AR signaling in androgen-sensitive prostate cancer cells. 

## 4. Materials and Methods

### 4.1. Test Compound

Lambertianic acid (purity: ≥97% as determined by HPLC) was kindly provided by Lee Min-Ho, college of the health industry, Eulji University (Seongnam City, Korea). The substance was analytically confirmed using NMR and GCMS by Eulji University.

### 4.2. Cell Culture Assay

LNCaP, PC-3 and DU145 cells were purchased from the Korea Cell Line Bank (Seoul, Korea) and maintained in RPMI1640 medium (10% FBS, 2 mM l-glutamine, 10 mM HEPES, 1 mM sodium pyruvate, 45 g/L glucose and penicillin/streptomycin) (WelGene, Daegu, Korea) in a cell incubator.

### 4.3. Cell Viability Assay

Cells (LNCaP, DU-145 and PC-3 cells, 1 × 10⁴ cells/well) were treated with various concentrations (0, 3.125, 6.25, 12.5, 25, 50, 100 μM and 200 μM) of LA in 96-well plates (SPL Life Science, Gyeonggi-do, Korea). After 24 h, 50 μL of MTT solution (1 mg/mL, Sigma, St. Louis, MO, USA) were added. After 1 h of incubation, optical density was determined by the microplate reader (Tecan, Switzerland) at 570 nm.

### 4.4. Western Blot Analysis 

LNCaP cells (5 × 10^5^ cells/well) were cultured with or without LA (50, 100 μM and 200 μM), mibolerone (Mib, 1 nM) or both in 6-well plates. Cell were lysed in radioimmunoprecipitation assay (RIPA) buffer (50 mM Tris-HCL, pH 7.4, 150 mM sodium chloride (NaCl), 1% NP-40, 0.25% deoxycholic acid-Na, 1 M EDTA, 1 mM sodium orthovanadate (Na_3_VO_4_), 1 mM sodium fluoride (NaF) and protease inhibitor cocktail). Protein samples were quantified by using a Bio-Rad DC protein assay kit II (Bio-Rad, Hercules, CA, USA), separated by electrophoresis on a 10% to 15% SDS-PAGE gel and electrotransferred onto a Bio Trace NT transfer membrane (Pall, Gelman Laboratory, Port Washington, NY, USA). After blocking (3% nonfat skim milk), the membrane probed with primary antibodies against AR (BD Biosciences), PSA (Dako), p53 (Santa Cruz Biotechnologies, Santa Cruz, CA, USA), p21 (Cell Signaling Technology), p27, cyclin D1, CDK4, CDK6, B-cell lymphoma 2 (Bcl-2), Bcl-2-associated X protein (BAX) (Santa Cruz Biotechnologies, Santa Cruz, CA, USA), cleaved caspase-3, cleaved caspase-9 (Cell Signaling Technology), poly-ADP-ribose polymerase (PARP, Santa Cruz Biotechnologies, Santa Cruz, CA, USA) and β-actin (Sigma-Aldrich) overnight and then exposed to HRP-conjugated secondary anti-mouse or rabbit antibodies. Protein expression was examined using the EZ-Western Lumi Pico (DOGEN).

### 4.5. Crystal Violet Staining

LNCaP cells (1 × 10^5^ cell/well) were seeded in 6-well plates and treated with or without of LA (50, 100 and 200 μM) for 3 and 5 days. Then, the cells were washed with PBS and fixed with 1% glutaraldehyde in PBS at 25 °C for 20 min, then stained with 0.05% crystal violet solution. The cells were then resolved in 70% ethanol, and the OD was measured using an ELISA reader at 570 nm.

### 4.6. Cell Cycle Analysis

Cells were fixed with 75% ethanol and resuspended in PBS with RNase (1 mg/mL) at 37 °C for 1 h and stained with propidium iodide (PI). The stained cells were analyzed for DNA content by FACS Calibur containing Cell-Quest Software (Becton-Dickinson, Heidelberg, Germany).

### 4.7. AR Gene Silencing

LNCaP cells (2 × 10^5^ cell/well) were seeded onto a 6-well plate and transfected with 100 nM of AR-specific control (CON) siRNA or siRNA (Bioneer, Daejeon, Korea) using INTERFER transfection reagent (Poly Plus, Illkirch, France) for 24 or 48 h. Then, the cells were treated with or without of LA (100 μM), and transfection was confirmed using Western blot analysis and a luciferase assay.

### 4.8. Luciferase Assay

LNCaP cells were transfected with luciferase reporter plasmids combined with a PSA-Luc reporter for 24 h, treated with LA for 24 h and then lysed followed by analysis of the reporter activity using the Luciferase Reporter Assay system (Promega, Madison, AL, USA).

### 4.9. Caspase-3 Activity

Caspase activity was measured using the manufacturer’s protocols (caspase-3 colorimetric assay kits; R&D Systems Inc., Minneapolis, MN, USA). Cells were lysed in lysis buffer. The cell lysates were treated with caspase-3-specific substrates at 37 °C for 4 h. Caspase-3 activity and absorbance were measured with a microplate reader at 430 nm.

### 4.10. Statistical Analysis

Data were shown as the means ± standard deviation (SD) of three or more replicates, and the statistical significance was verified by Student’s *t*-test.

## Supplementary Materials

Supplementary materials can be found at http://www.mdpi.com/1422-0067/17/7/1066/s1.

## Abbreviations

LA: lambertianic acid
AR: androgen receptor
PBS: phosphate-buffered saline
OD: optical density
