# Supplementary material for: Anti-Cancer Effect of Lambertianic Acid by Inhibiting the AR in LNCaP Cells
Source: Int J Mol Sci. 2016 Jul 7;17(7):1066. doi: 10.3390/ijms17071066 (PMC4964442; doi:10.3390/ijms17071066)
Supplement: Supplementary file 1 [file ijms-17-01066-s001.pdf]

Supplementary Materials: Anti-Cancer Effect of Lambertianic Acid by Inhibiting the AR in LNCaP Cells

Myoung-Sun Lee, Seon-Ok Lee, Sung-Hoon Kim, Eun-Ok Lee and Hyo-Jeong Lee

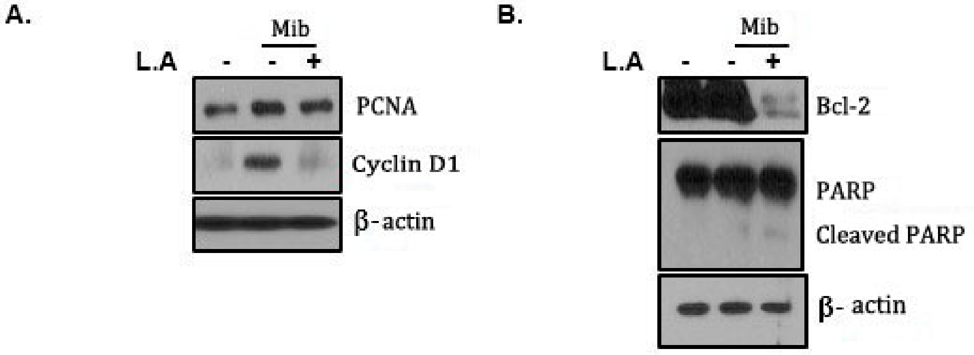

Figure S1. Anti-proliferative and apoptotic effect of Lambertianic acid on Mib-induced LNCaP cells. LNCaP cells were seeded in phenol red-free medium supplemented 5% charcoal-stripped serum. (**A**) Western blot analyses of PCNA and Cyclin D1 following treatment with LA, with or without mibolerone (Mib) for 24 h; (**B**) Western blot analyses of Bcl-2 and cleaved PARP following treatment LA for 48 h.
